# From Spike to Symptom: A Contribution to Decoding the Molecular Mechanisms Underlying SARS-CoV-2 Transmission, Infection, and Disease Pathology

**DOI:** 10.3390/ijms241512294

**Published:** 2023-08-01

**Authors:** João R. Mesquita

**Affiliations:** 1 ICBAS-School of Medicine and Biomedical Sciences, Porto University, 4050-313 Porto, Portugal; jrmesquita@icbas.up.pt; Tel.: +351-220428000; 2 Epidemiology Research Unit (EPIunit), Institute of Public Health, Porto University, 4050-600 Porto, Portugal; 3 Laboratório para a Investigação Integrativa e Translacional em Saúde Populacional (ITR), 4050-600 Porto, Portugal

## 1. Introduction

In December 2019, a novel coronavirus, SARS-CoV-2, was identified in Wuhan, China, from patients with severe pneumonia of unknown origin. This marked the beginning of the COVID-19 pandemic, a global health crisis that has had extremely significant social, economic, and public health implications. The scientific understanding of SARS-CoV-2 and COVID-19 has evolved rapidly since its discovery, with researchers and healthcare professionals worldwide working together to comprehend the virus and develop effective strategies to control its spread [1]. Coronaviruses belong to the *Coronaviridae* family and are enveloped, positive-sense, single-stranded RNA viruses. These viruses are known to infect various animal species and occasionally cross the species barrier to infect humans. The first known coronavirus outbreak in humans was the Severe Acute Respiratory Syndrome (SARS) epidemic in 2002–2003, caused by the SARS-CoV virus. Subsequently, the Middle East Respiratory Syndrome (MERS) outbreak in 2012 was caused by MERS-CoV [2].

SARS-CoV-2 shares significant genetic similarity with SARS-CoV and is classified as a member of the betacoronavirus genus. However, there are important differences between the two viruses, particularly in their transmissibility and pathogenicity. SARS-CoV-2 is highly transmissible, primarily through respiratory droplets, which has contributed to its rapid global spread. The primary route of transmission occurs when an infected person exhales respiratory droplets containing the virus, which can be inhaled by others in close proximity. Additionally, the virus can be contracted by touching surfaces contaminated with the virus and then touching the face, particularly the eyes, nose, or mouth. Asymptomatic carriers can also spread the virus, making it challenging to control its transmission [3].

COVID-19 can lead to a wide range of symptoms, from mild or asymptomatic cases to severe respiratory distress and multi-organ failure. Common symptoms include fever, cough, shortness of breath, fatigue, loss of taste or smell, and muscle aches. Severe cases may progress to acute respiratory distress syndrome, which can be fatal [1].

The COVID-19 pandemic has prompted an unprecedented global effort in scientific research, vaccine development, and public health measures. Governments, researchers, and pharmaceutical companies have collaborated to develop and deploy effective vaccines to prevent COVID-19. Several vaccines, based on different technologies, have been authorized for emergency use or fully approved by various regulatory agencies. These vaccines have played a crucial role in reducing the severity and spread of the virus.

The molecular aspects of SARS-CoV-2 viral transmission involve a complex interplay of viral proteins, host cell receptors, and immune responses. Understanding these processes is critical for developing targeted interventions to control the spread of the virus and prevent COVID-19. Researchers continue to investigate the molecular mechanisms of SARS-CoV-2 transmission to identify potential therapeutic targets and further enhance our understanding of this virus’s behavior. However, a few years have elapsed since the discovery of SARS-CoV-2, and much information is still needed on the molecular aspects of viral transmission. Hence, a molecular approach to SARS-CoV-2 transmission is of the utmost importance.

This Special Issue is a continuation of the Special Issue “Molecular Advances in SARS-CoV-2 Transmission, Infection, and Pathology”, and presents a compilation of recent advances in the molecular detection and pathology of SARS-CoV-2. Original research articles and comprehensive reviews that cover the molecular aspects of SARS-CoV-2 transmission, infection, and pathology were included in this Special Issue, in a total of nine papers.

## 2. Reviews on Molecular Mechanisms, Pathogenesis and Markers

It first starts with four interesting reviews, the first entitled “Molecular Mechanisms Responsible for Diabetogenic Effects of COVID-19 Infection—Induction of Autoimmune Dysregulation and Metabolic Disturbances” aimed at elucidating the intricate molecular mechanisms that underlie the diabetogenic effects of COVID-19 by exploring the potential involvement of various factors, including direct damage to 𝛽-cells, insulin resistance triggered by systemic inflammation, and disruptions in hormonal regulation. The binding of SARS-CoV-2 to angiotensin-converting enzyme 2 receptors, which are present in key metabolic organs and tissues, is postulated to perturb glucometabolic pathways, leading to hyperglycemia and potentially contributing to new disease mechanisms. Moreover, the virus’s impact on 𝛽-cells, whether through direct invasion or systemic inflammation, may induce insulin resistance and disrupt glucose homeostasis. Additionally, glucocorticoids, commonly used in COVID-19 treatment, may exacerbate hyperglycemia and insulin resistance, potentially leading to new-onset diabetes. As such, the authors provide a comprehensive overview of the current understanding of the interplay between COVID-19 and diabetes, emphasizing potential areas for future research and therapeutic interventions, ultimately increasing the comprehension of how COVID-19 impacts the development and progression of diabetes.

The second review entitled “Possible Pathogenesis and Prevention of Long COVID: SARS-CoV-2-Induced Mitochondrial Disorder” focuses on the fact that patients who have recuperated from COVID-19 may encounter persistent exhaustion during exercise, despite the absence of evident heart or lung abnormalities, with the existing dearth of effective treatments posing a significant challenge in managing long COVID. The authors hypothesize that one potential underlying mechanism of long COVID could involve mitochondrial dysfunction by SARS-CoV-2 infection modifying the mitochondria responsible for cellular energy production, which in turn could lead to dysfunctional mitochondria, heightened oxidative stress, and a subsequent loss of mitochondrial integrity and cell death. Furthermore, there is evidence that viral proteins may interact with mitochondrial complexes, disrupting mitochondrial function and triggering exaggerated immune cell responses. These responses result in inflammation and potentially contribute to long COVID-19 symptoms. Importantly, the authors also note that it is crucial to acknowledge that the precise roles of mitochondrial damage and inflammatory responses induced by SARS-CoV-2 in the development of long COVID are still being unraveled and assure further studies on the topic.

The third review entitled “COVID-19 Biomarkers at the Crossroad between Patient Stratification and Targeted Therapy: The Role of Validated and Proposed Parameters” is focused on the argument that due to the diverse range of COVID-19 presentations, accurately stratifying patients upon admission remains challenging, posing difficulties in the rational allocation of limited medical resources and in implementing personalized therapeutic approaches. The authors highlight that numerous hematologic biomarkers have been validated not only to aid in the early triage of SARS-CoV-2-positive patients and to monitor disease progression, but some even represent potential targets for direct or indirect pharmacological intervention, offering the possibility of a more customized treatment approach, particularly for those with severe and progressive disease. In this narrative review, the authors provided an overview of the most commonly used biomarkers in clinical practice and highlight the most promising ones identified in specific population studies. Lastly, the authors conclude that integrating new and highly informative markers into routine clinical testing could aid in early patient stratification and guide timely and tailored therapeutic interventions for improved patient outcomes.

The fourth review, entitled “Nanoparticles in Clinical Trials: Analysis of Clinical Trials, FDA Approvals and Use for COVID-19 Vaccines”, starts by highlighting the applications of nanoparticles not only in various medical domains, but notably in drug delivery, offering numerous advantages as drug carriers, including reduced toxicity, improved bioavailability, and the potential for targeted modifications to specific tissues or cells. As a baseline example, authors state that lipid-based nanoparticles have played a pivotal role in the development and production of widely used COVID-19 vaccines. In this review, the authors present a quantitative analysis of clinical trials involving nanoparticles conducted from 2002 to 2021, along with recently FDA-approved drugs (since 2016). From a total of 486 clinical trials identified, the authors state that liposomes (44%) and protein-based formulations (26%) were the dominant types of nanoparticles investigated during this period. The most commonly studied nanoparticle contents included paclitaxel (23%), metals (11%), doxorubicin (9%), bupivacaine, and various vaccines (both at 8%). Regarding FDA-approved nanoparticle drugs, polymeric (29%), liposomal (22%), and lipid-based (21%) drugs constituted the most prevalent categories. Finally, authors also explore the differential progress of distinct nanoparticle groups and their contents, as well as the underlying factors contributing to these trends.

## 3. Diverse Array of Original Research Contributions: An Exploration of Varied Scientific Studies

The Special Issue concludes with five cutting edge research papers covering a wide array of topics within the SARS-CoV-2 area and, in particular, in the molecular mechanisms underlying SARS-CoV-2 transmission, infection, and disease pathology.

The first research study, entitled “Comparative Analysis of Library Preparation Approaches for SARS-CoV-2 Genome Sequencing on the Illumina MiSeq Platform”, first highlights that since the start of the COVID-19 pandemic, the continuous emergence of new variants and successive waves of the virus have posed significant challenges and genomic surveillance has proven to be a critical tool in studying viral circulation. Numerous technological approaches are currently employed for the whole genome sequencing (WGS) of SARS-CoV-2 and the published study focuses on the comparison of three different protocols for constructing SARS-CoV-2 WGS libraries: an amplicon-based protocol utilizing a commercial primer panel, an amplicon-based protocol using a custom panel, and a hybridization capture protocol. By analyzing these protocols, the researchers identified specific disparities in sequencing depth, genomic coverage, and the number of single-nucleotide polymorphisms (SNPs) detected, with the custom panel-based approach demonstrating favorable outcomes and providing a predictable output that proves valuable for the epidemiological surveillance of SARS-CoV-2 variants.

The second research study entitled “T-Cell Immunity in COVID-19-Recovered Individuals and Individuals Vaccinated with the Combined Vector Vaccine Gam-COVID-Vac” conducted research on T-cell immunity in individuals who had recovered from mild and moderate COVID-19 and in those vaccinated with the Gam-COVID-Vac combined vector vaccine. The authors used the ELISPOT method to assess the number of T-cells responding with IFN-γ synthesis upon stimulation by peptides containing epitopes of the S-protein or N-, M-, ORF3, and ORF7 proteins, employing peripheral blood mononuclear cells. The percentage of positive determinations concerning the T-cell immune response to SARS-CoV-2 antigens in control, recovered, and vaccinated individuals was 12%, 70%, and 52%, respectively, and over half of the vaccinated individuals with a T-cell response displayed sensitivity to antigens from N-, M-, ORF3, and ORF7 proteins, which are not produced by Gam-COVID-Vac, suggesting a substantial likelihood of asymptomatic SARS-CoV-2 infection. Furthermore, the enhanced release of IFN-γ by individual sensitized T-cells in response to specific stimulation in recovered and vaccinated individuals did not lead to an accumulation of this or other cytokines in the culture medium. In all, findings indicated that there is a delicate balance between cytokine production and utilization by immunocompetent cells, which is crucial for providing a controlled cytokine signal and preventing a “cytokine storm”.

The third research study, entitled “Prediction of Recurrent Mutations in SARS-CoV-2 Using Artificial Neural Networks”, focuses on the prominent issue that is the prediction of SARS-CoV-2 mutations. The authors highlight that predicting recurrent mutations that are driven by the host is key, particularly those caused by host deaminases. In the study, they utilized machine learning techniques to forecast which positions in the SARS-CoV-2 genome are likely to undergo recurrent mutations and which specific mutations are most likely to occur frequently. They divided their data in three distinct sets: a training set, a validation set, and an independent test set and their prediction model achieved promising results when evaluated on the test set, with a specificity value of 0.69, a sensitivity value of 0.79, and an Area Under the Curve of 0.8, indicating the feasibility of accurately predicting recurrent SARS-CoV-2 mutations. For validation purposes, they compared their results with posterior data, and the analysis revealed that some of the false positive predictions were later verified as true positive mutations, reinforcing the reliability of the proposed approach. They then expanded the investigation to analyze mutations associated with variants of concern, such as Alpha, Beta, Delta, Gamma, and Omicron and examined predicted recurrent mutations in the M-protein and spike proteins of the virus.

The fourth research study entitled “Cytokine Profiling in Different SARS-CoV-2 Genetic Variants” presents the continuation of the authors’ previous research on the alterations in the chemokine profile linked to various genetic variants of SARS-CoV-2 during infection. Authors assessed cytokine concentrations in COVID-19 patients infected with distinct SARS-CoV-2 variants, including the ancestral Wuhan strain, Alpha, Delta, and Omicron variants, thereby considering both the viral and host immune responses. A total of 340 biological samples from COVID-19 patients and healthy donors were subjected to genotyping of the virus, followed by cytokine concentration analysis in blood plasma. Results indicated that among the 30 studied cytokines, only 4 exhibited sustained elevation independent of the viral variant (IL-6, IL-10, IL-18, and IL-27). The authors designated them as putative ‘constant’ markers for COVID-19 infection. In all, the authors concluded that their results were preliminary but interesting, and advocated further investigation and comparative analysis of immune responses elicited by diverse SARS-CoV-2 variants.

The fifth and final research study, entitled “A Comparative Study of the Plasma Chemokine Profile in COVID-19 Patients Infected with Different SARS-CoV-2 Variants”, starts by recognizing the pivotal role that chemokines play in orchestrating the recruitment, proliferation, and activation of immune cells during the COVID-19 inflammatory response. To clarify this interplay, the authors quantified and analyzed the concentrations of different chemokines implicated in COVID-19, which arise from distinct variants of the virus. They measured the concentrations of 11 chemokines in collected samples, namely CCL2/MCP-1, CCL3/MIP-1α, CCL4/MIP-1β, CCL7/MCP-3, CCL11/Eotaxin, CCL22/MDC, CXCL1/GROα, CXCL8/IL-8, CXCL9/MIG, CXCL10/IP-10, and CX3CL1/Fractalkine, and found statistically significant elevations in the concentrations of CCL2/MCP-1, CXCL8/IL-8, and CXCL1/IP-10 across all viral variants studied, while a decline was observed in the concentrations of CCL22/MDC. This suggests that viral protein mutations may play a crucial role in modulating cellular and molecular mechanisms involved in immune responses, and authors the underscore the importance of understanding the impact of viral variants on immune responses and disease outcomes in COVID-19.

## 4. Concluding Remarks

This is a short and meaningful collection of studies that provide an overview of the molecular aspects of SARS-CoV-2 viral transmission and the complex interplay of viral proteins, host cell receptors, and immune responses. The global perspective highlighted by the content of this collection reinforces the need to develop targeted interventions to control the spread of the virus and prevent COVID-19.
